# Outcomes of Harmonic Scalpel and Electrocautery in Patients Who Underwent Modified Radical Mastectomy

**DOI:** 10.7759/cureus.12311

**Published:** 2020-12-26

**Authors:** Farhana Memon, Ashfaque Ahmed, Sughra Parveen, Sadaf Iqbal, Adnan Anwar, Atif A Hashmi

**Affiliations:** 1 General Surgery, Sir Syed Medical College, Karachi, PAK; 2 General Surgery, Civil Hospital, Karachi, PAK; 3 General Surgery, Jinnah Postgraduate Medical Centre, Karachi, PAK; 4 General Surgery, Baqai Medical University, Karachi, PAK; 5 Physiology, Al-Tibri Medical College, Isra University, Karachi, PAK; 6 Pathology, Liaquat National Hospital and Medical College, Karachi, PAK

**Keywords:** harmonic scalpel, modified radical mastectomy, electrocautery, breast cancer

## Abstract

Objective

This study aimed to compare the mean operative time, total analgesic required, and the mean number of drainage days in harmonic scalpel versus electrocautery in breast cancer patients undergoing modified radical mastectomy (MRM).

Methodology

This retrospective cross-sectional study was conducted in the Department of General Surgery, Jinnah Postgraduate Medical Center (JPMC). The duration of the study was six months, from January 2018 until July 2018. A total of 194 females with biopsy-proven breast cancer undergoing MRM were included in the study. They were divided into two groups. In group ‘A', a harmonic scalpel was used, and in group 'B', electrocautery was used for hemostasis.

Results

The mean age of the participants was 48.68 ±10.04 years. The mean operative time was 102.13 ±2.04 minutes. The mean number of days of drainage was 1.27 ±2.63. The mean analgesia amount was 30.72 ±3.25 mg. In the harmonic scalpel group, the mean operative time was 100.43 ±0.89 minutes, whereas, in the electrocautery group, it was 103.86 ±1.12 minutes with a significant difference (p=0.001). In the harmonic scalpel group, the mean number of drainage days was 8.90 ±0.42, whereas, in the electrocautery group, it was 13.58 ±1.26 with a statistically significant difference (p=0.001). In the harmonic scalpel group, the mean analgesia amount was 1,800.5 ±353.55 mg, whereas, in the electrocautery group, it was noted to be 2,006.25 ±289.43 with a statistically significant difference (p=0.001).

Conclusion

This study concludes that compared with standard electrocautery, harmonic scalpel dissection is associated with significant benefits in decreasing postoperative drainage and blood loss during operations after MRM for breast cancer.

## Introduction

The most common type of cancer affecting females is breast cancer [1]. It is the most widespread malignancy, and it affects 14% of the female population globally [2]. The occurrence of breast cancer has increased significantly in developing countries [3,4]. The appearance of a new bulge or mass is the most common sign of breast cancer [5]. Other signs of breast cancer are swelling of either the whole breast or a portion of the affected breast, dimpling of the skin associated with nipple pain or an inverted nipple, marked redness in the breast, thickening of the skin over the nipple, discharge from the nipple, and a mass felt in the axilla [6]. Advanced age and female sex are the main risk factors for breast cancer. Other risk factors include genetics, hormonal changes, certain dietary habits, lack of childbearing or lack of breastfeeding, and being overweight [7].

Several diagnostic procedures are used for the diagnosis of breast cancers. A lump or mass is felt on touch during a physical examination of breasts by either the person itself or the healthcare practitioner. A patient normally undergoes breast mammography after the confirmation of the presence of a mass or lump in the breast. The mammography is performed to visualize the compact and thickened areas of breast tissues, which can then diagnose a breast lump as either malignant or benign. Fine-needle aspiration cytology (FNAC) or trucut biopsy is used to confirm the diagnosis of malignancy. Early diagnosis and treatment of breast cancer increase the chances of complete healing [8].

Electrocautery is the most frequently used surgical procedure for the dissection and hemostasis in modified radical mastectomy (MRM), and it is used way more frequently than conventional scalpel [9]. However, past studies have proven that electrocautery is associated with increased postoperative complications such as seroma, wound infection, flap necrosis, hematoma, and a longer duration of drainage that interferes with adjuvant treatments following the operation [10]. The harmonic scalpel is used nowadays in laparoscopic surgeries and for dissection in MRM. High-frequency mechanical vibrations are used in a harmonic scalpel, in which cutting and coagulation happen simultaneously, with a comparatively low temperature resulting in fewer thermal injuries compared to electrocautery [11]. The biomechanics of harmonic scalpel entails ultrasonic vibrations inactivating protein in which a high frequency of 55,500 Hz is applied with a vibratory excursion of 50-100 μm [12].

Harmonic scalpel helps reduce the operative time and minimize blood loss, drainage volume, and development of seroma and complications of wound compared with electrocautery, as reported by other published studies [13,14]. Hence, this study aimed to assess the outcomes of patients undergoing harmonic scalpel versus electrocautery in the treatment of breast cancer.

## Materials and methods

This retrospective observational study was conducted in the Department of General Surgery, Jinnah Postgraduate Medical Center (JPMC), Karachi. The period of the study was six months, from January 2018 until July 2018. The total sample size was 194; female patients aged between 18 and 65 years who were diagnosed with stage II and III breast cancer on biopsy and underwent MRM were enrolled in the study. They were divided into two groups. In group ‘A', the harmonic scalpel was used, and in group 'B', electrocautery was used. The exclusion criteria for this study were as follows: patients in early stages of breast cancer (T1), those who had undergone breast surgery in the past, patients with no adjuvant therapy, those with metastasis, diabetes, and other comorbidities like blood diseases, patients who were not willing to undergo the procedures, and those who were not fit for general anesthesia.

Operative time was defined as the time from skin incision to the last stitch for skin closure. Analgesics were used if the patients complained of pain of more than 3 points as per the visual analog scale (VAS) score. The amount of analgesia used at the end of 36 hours was noted and days of drainage were noted at the end of the seventh postoperative day. Blood loss during the operation was measured by the amount of blood collected in the suction apparatus.

The data were entered and analyzed on SPSS Statistics version 26.0 (IBM Inc., Armonk, NY). Mean ±SD was computed for age, operative time, days of drainage, and the amount of analgesia needed in the two groups. Frequencies and percentages were computed for the stage of the disease. An independent t-test was applied to check the associations, and p-values of ≤0.05 were considered statistically significant.

## Results

The total number of females enrolled in the study was 194. The mean age of the patients was 48.68 ±10.04 years. The mean operative time was 102.13 ±2.04 minutes. The mean number of drainage days was 1.27 ±2.63. The mean analgesia amount was 30.72 ±3.25 mg. Most of the patients (162, 83.5%) were above 40 years of age (Table 1).

**Table 1:** Age distribution among breast carcinoma patients and patients' record (n=194) SD: standard deviation

Variable		Values
Age of the patients (in years), mean ±SD		48.68 ±10.04
Age group	≤40 years, n (%)	32 (16.5%)
	>40 years, n (%)	162 (83.5%)
Operative time (minutes), mean ±SD		102.13 ±2.04
Days of drainage, mean ±SD		1.27 ±2.63
Analgesia amount (mg), mean ±SD		30.72 ±3.25

In the harmonic scalpel group, stage II disease was found in 65 (67%) patients and stage III was found in 32 (33%) patients. In the electrocautery group, stage II disease was found in 64 (66%) patients and stage III disease was found in 33 (34%) patients (Table 2, Table 3).

In the harmonic scalpel group, the mean operative time was found to be 100.43 ±0.89 minutes, whereas, in the electrocautery group, the mean operative time was found to be 103.86 ±1.12 minutes (p=0.001). In the harmonic scalpel group, the mean number of days of drainage was found to be 8.90 ±0.42, whereas the number of mean days of drainage in the electrocautery group was found to be 13.58 ±1.26 (p=0.001). In the harmonic scalpel group, the mean analgesia amount was found to be 1,800.5 ±353.55 mg, whereas, in the electrocautery group, the mean analgesia amount was found to be 2,006.25 ±289.43 mg (p=0.001) (Table 2).

**Table 2:** Comparison of groups with respect to operative time, drainage days, and analgesia required in stage II breast carcinoma patients (n=129) SD: standard deviation

Variables	Groups A and B (n)	Mean ±SD	P-value
Operative time (minutes)	Harmonic scalpel, group A (65)	100.43 ±0.89	0.001
	Electrocautery, group B (64)	103.86 ±1.12	
Drainage days	Harmonic scalpel (65)	8.90 ±0.42	0.001
	Electrocautery (64)	13.58 ±1.26	
Analgesia amount (mg)	Harmonic scalpel (65)	1,800.5 ±353.55	0.001
	Electrocautery (64)	2,006.25 ±289.43	

For patients with stage III breast cancer, in the harmonic scalpel group, the mean operative time was found to be 100.10 ±0.001 minutes, whereas, in the electrocautery group, the mean operative time was found to be 104.07 ±1.08 minutes (p=0.001). In the harmonic scalpel group, the mean number of drainage days was found to be 8.91 ±0.46, whereas, the mean number of drainage days in the electrocautery group was found to be 13.72 ±2.03 (p=0.001). In the harmonic scalpel group, the mean analgesia amount was found to be 1,800 ±101.60 mg, whereas, in the electrocautery group, the mean analgesia amount was found to be 2,036.36 ±365.56 mg (p=0.001) (Table 3).

**Table 3:** Comparison of groups with respect to operative time, drainage days, and analgesia required in stage III breast carcinoma patients (n=65) SD: standard deviation

Variables	Groups A and B (n)	Mean ±SD	P-value
Operative time (minutes)	Harmonic scalpel, group A (32)	100.10 ±0.001	0.001
	Electrocautery, group B (33)	104.07 ±1.08	
Drainage days	Harmonic scalpel (32)	8.91 ±0.46	0.001
	Electrocautery (33)	13.72 ±2.03	
Analgesia amount (mg)	Harmonic scalpel (32)	1,800 ±101.60	0.001
	Electrocautery (33)	2,036.36 ±365.56	

## Discussion

In the present study, we found that harmonic scalpel dissection had significant benefits over standard electrocautery in terms of operative time, drainage days, and analgesia amount in patients undergoing MRM for breast cancer. 

Choosing harmonic scalpel dissection helps to reduce the drainage time after the operation, the development of seroma, intraoperative bleeding, and the procedure also reduces the complications of wounds in MRM. It has the advantage that it decreases operating time compared with electrocautery.

In another study about the evaluation of the efficacy of harmonic scalpel versus electrocautery in patients undergoing MRM, it was proven that outcomes of MRM by using harmonic scalpel had a more positive impact than electrocautery [15]. These findings were consistent with the findings of our study.

In another study, 62% of the patients were found to be in the age group of 40-59 years. Statistically, it is believed that middle-aged women have a higher chance of developing breast cancer. In that study, the mean age of the patients in the harmonic scalpel group was 50.36 ±11.04 years, and that of patients in the electrocautery group was 52 ±11.19 years [16]. Our study findings were also consistent with the previous study in that they showed a higher risk of breast carcinoma in middle-aged women; the mean age of the patients who underwent MRM in our study was 48.68 ±10.04 years. Other studies have also found that the frequency of disease was most common in middle-aged women [17,18]. The disease's age-related incidence rises until menopause and increases more slowly subsequently [19]. In our study, 32 (16.5%) patients with breast cancer were less than 40 years of age, while 162 (83.5%) were more than 40 years of age.

The findings of our study revealed that the mean operative time was 100.32 ±0.74 minutes in the harmonic scalpel group, whereas, in the electrocautery group, the mean operative time was 103.93 ±1.11 minutes (p=0.001). In the harmonic scalpel group, the mean number of drainage days was 1.90 ±0.43, whereas the mean number of drainage days in the electrocautery group was 2.63 ±1.55 (p=0.001). However, the results of our study contrast with the results of Khan et al., who reported that the harmonic scalpel does not reduce the operative time but reduces postoperative distress and morbidity [20].

Drains are positioned to help for checking complications such as hematoma, seroma, and flap necrosis [21]. AbulNagah et al. [22] have observed that the time required for axillary dissection was less in patients operated by a harmonic scalpel compared to those who underwent electrocautery (mean: 18 ±3.11, p=0.004). The authors also proved that the postoperative blood loss (ml) was lower in the harmonic scalpel group compared to the electrocautery group (mean: 212.75 ±117.24 vs. 357 ±172.71, p=0.003). Furthermore, the mean total-draining volume in the harmonic scalpel was 838 ±473.93 ml, while the draining volume in the electrocautery was 1,312.75 ±823.79 ml (p=0.02). Results of that study were consistent with our study; we observed that the mean operative time with the harmonic scalpel was 100.43 ±0.89 (p=0.001) and the mean number of drainage days in the harmonic scalpel group was 8.90 ±0.42, which was significantly less compared to electrocautery.

Our findings were consistent with the study by Parveen et al. [23], which showed that the intraoperative blood loss with the harmonic scalpel was 45 ±12 ml, the operative time was 90 ±7 minutes, the drainage volume of flap drain and the axillary drain was 20 ±2.0 ml and 155 ±35 ml respectively, and the drainage duration was 1.3 ±0.2 and 2.7 ±0.5 days for the flap and axillary drain respectively.

Another study by Deo and Shukla [9] demonstrated a significant reduction in drainage days in the harmonic scalpel group compared to the electrocautery group. Studies by Khan et al. [20] and Huang et al. [24] have also shown a reduction in the drainage days with the use of a harmonic scalpel. Our study findings were also consistent with this study as our study revealed that the number of drainage days was fewer in the harmonic scalpel group (8.91 ±0.46) compared to the electrocautery group (13.72 ±2.03).

The quantitative approach of our study has ensured that we have sampled an extensive range of females with breast cancers. However, the study is not free from selection and practice bias. Considering the observations made in our study and assessing as to what extent they may be associated with the other treatment modalities would prove beneficial in discovering more facts about the treatment of breast cancer.

## Conclusions

Based on our findings, the procedure with harmonic scalpel showed much better results and outcomes in terms of mean operative time, the requirement of total analgesic, and the mean number of drainage days compared to electrocautery in patients undergoing MRM. It also proved to be more cost-effective as it led to shorter hospital stays since it made early discharge possible thanks to early drain removal and reduction in the dose of analgesia required.

## References

[1] Hashmi AA, Edhi MM, Naqvi H, Khurshid A, Faridi N (2014). Molecular subtypes of breast cancer in South Asian population by immunohistochemical profile and Her2neu gene amplification by FISH technique: association with other clinicopathologic parameters. Breast J.

[2] Jemal A, Bray F, Center MM, Ferlay J, Ward E, Forman D (2011). Global cancer statistics. CA Cancer J Clin.

[3] Hashmi AA, Edhi MM, Naqvi H, Faridi N, Khurshid A, Khan M (2014). Clinicopathologic features of triple negative breast cancers: an experience from Pakistan. Diagn Pathol.

[4] Hashmi AA, Naz S, Hashmi SK (2019). Epidermal growth factor receptor (EGFR) overexpression in triple-negative breast cancer: association with clinicopathologic features and prognostic parameters. Surg Exp Pathol.

[5] Giuliano AE, Hunt KK, Ballman KV (2011). Axillary dissection vs no axillary dissection in women with invasive breast cancer and sentinel node metastasis: a randomized clinical trial. JAMA.

[6] Kuraparthy S, Reddy KM, Yadagiri LA, Yutla M, Venkata PB, Kadainti SV, Reddy RP (2007). Epidemiology and patterns of care for invasive breast carcinoma at a community hospital in Southern India. World J Surg Oncol.

[7] Hayes J, Richardson A, Frampton C (2013). Population attributable risks for modifiable lifestyle factors and breast cancer in New Zealand women. Intern Med J.

[8] Saslow D, Hannan J, Osuch J (2004). Clinical breast examination: practical recommendations for optimizing performance and reporting. CA Cancer J Clin.

[9] Deo SV, Shukla NK (2000). Modified radical mastectomy using harmonic scalpel. J Surg Oncol.

[10] Yilmaz KB, Dogan L, Nalbant H, Akinci M, Karaman N, Ozaslan C, Kulacoglu H (2011). Comparing scalpel, electrocautery and ultrasonic dissector effects: the impact on wound complications and pro-inflammatory cytokine levels in wound fluid from mastectomy patients. J Breast Cancer.

[11] Galatius H, Okholm M, Hoffmann J (2003). Mastectomy using ultrasonic dissection: effect on seroma formation. Breast.

[12] Foschi D, Cellerino P, Corsi F, Taidelli T, Morandi E, Rizzi A, Trabucchi E (2002). The mechanisms of blood vessel closure in humans by the application of ultrasonic energy. Surg Endosc.

[13] Kozomara D, Galić G, Brekalo Z, Sutalo N, Kvesić A, Soljić M (2010). A randomised two-way comparison of mastectomy performed using harmonic scalpel or monopolar diathermy. Coll Antropol.

[14] Rohaizak M, Khan FJ, Jasmin JS, Mohd Latar NH, Abdullah SS (2013). Ultracision versus electrocautery in performing modified radical mastectomy and axillary lymph node dissection for breast cancer: a prospective randomized control trial. Med J Malaysia.

[15] Kontos M, Kothari A, Hamed H (2008). Effect of harmonic scalpel on seroma formation following surgery for breast cancer: a prospective randomized study. J BUON.

[16] Khater A (2010). Harmonic scalpel as a single instrument in modified radical mastectomy. Is it more cost effective than electrocautery and ligature. Egypt J Surg.

[17] Hashmi AA, Naz S, Hashmi SK (2018). Prognostic significance of p16 & p53 immunohistochemical expression in triple negative breast cancer. BMC Clin Pathol.

[18] Hashmi AA, Naz S, Hashmi SK (2018). Cytokeratin 5/6 and cytokeratin 8/18 expression in triple negative breast cancers: clinicopathologic significance in South-Asian population. BMC Res Notes.

[19] Benz CC (2008). Impact of aging on the biology of breast cancer. Crit Rev Oncol Hematol.

[20] Khan S, Khan S, Chawla T, Murtaza G (2014). Harmonic scalpel versus electrocautery dissection in modified radical mastectomy: a randomized controlled trial. Ann Surg Oncol.

[21] Kuroi K, Shimozuma K, Taguchi T, Imai H, Yamashiro H, Ohsumi S, Saito S (2006). Evidence-based risk factors for seroma formation in breast surgery. Jpn J Clin Oncol.

[22] AbulNagah G, EL-Fayoumi T, Lotfy H, Shehab W, Tarek A (2007). Comparative study between using harmonic scalpel and Electrocautery in modified radical mastectomy. Egypt J Surg.

[23] Parveen S, Qureshi S, Sarwar 0, Damani SR (2012). Modified radical mastectomy with axillary clearance using harmonic scalpel. Pak J Surg.

[24] Huang J, Yu Y, Wei C, Qin Q, Mo Q, Yang W (2015). Harmonic scalpel versus electrocautery dissection in modified radical mastectomy for breast cancer: a meta-analysis. PLoS One.

